# Maximizing quinoa production through a dual-purpose harvesting method

**DOI:** 10.3389/fpls.2025.1606163

**Published:** 2025-07-16

**Authors:** Grato Ndunguru, Addissu G. Ayele, Safiullah Pathan

**Affiliations:** Department of Agriculture and Environmental Sciences, Lincoln University of Missouri, Jefferson City, MO, United States

**Keywords:** quinoa, dual-purpose, nutritional composition, grains, leafy green, planting date

## Abstract

This study introduces a novel dual-purpose quinoa production method, allowing the harvest of leafy greens first and grains later from the same plant, thereby enhancing overall production and economic returns. Four quinoa lines were evaluated under two treatments: (1) cut, where leafy greens were harvested four weeks after germination, and plants were subsequently allowed to mature, and (2) control, where plants were grown to full maturity without cutting. The study employed a randomized complete block design (RCBD) with three replications across three planting dates spaced at one-week intervals. Similar management practices were followed for both treatments and the date of planting. Results showed a significantly higher grain yield in cut plants (22.87g plant^-1^) compared to control plants (15.52g plant^-1^, *p* < 0.05). Quinoa line PI614927 yielded the highest in both cut and control treatments, with 29.15 and 18.33g plant^-1^, respectively. Grain yield was higher in the cut than the control on all three planting dates. The cut plants matured two days later than the control due to late branching and flowering. Shoot dry weight was significantly higher in cut plants (77.67g) than in the control (53.80g plant^-1^) due to a higher number of branches in cut plants (4–6 plant^-1^). In contrast, plant height and panicle length were higher in the control plant. The root dry weight was significantly higher in the cut (14.99g) than in the control (12.87g plant^-1^) plants. The two treatments showed no significant differences in 100-seed weight and root length. There was no significant difference in the nutritional compositions of quinoa grains between the treatments and dates of planting. This study has three benefits: harvesting leafy greens and higher grains, profound environmental benefits from using less water and fertilizer, one-time field preparation, and short duration (around 100 days). These research findings can accelerate quinoa leafy greens and grains production, contributing to food and nutritional security and sustainable alternative crop production, especially for small farmers.

## 1 Introduction

Quinoa (*Chenopodium quinoa* Willd.) is a nutrient-rich and abiotic stress-resilient crop of the Andean regions of South America, domesticated about 7,000 years ago, mainly in Lake Titicaca between Peru and Bolivia. The Incas honored it as a sacred grain and nicknamed it ‘chisya mama’, which means ‘mother grain’ (Bazile et al., 2016; Walters et al., 2016; Jacobsen, 2017). Once neglected and underutilized, quinoa has recently been rediscovered and has regained popularity worldwide due to its outstanding nutritional quality, human health benefits, and adaptability to diverse environments. Quinoa is a gluten-free grain with a low glycemic index and contains high protein levels, essential amino acids, important minerals, and vitamins (Bazile and Baudron, 2015; Bazile et al., 2016; Gordillo-Bastidas et al., 2016). Moreover, quinoa is rich in bioactive components and offers health-promoting properties, including antimicrobial, anticancer, antidiabetic, and anti-obesity, cardiovascular protection benefits (Villacrés et al., 2022; Huang et al., 2024; Xi et al., 2024). Due to these nutritional properties and health benefits, quinoa is considered a novel, functional, and popular health food – often referred to as a ‘superfood.’ Considering its importance, the United Nations (UN) acknowledged 2013 as the ‘International Year of Quinoa.’ In 2022, global quinoa production was 158.98 thousand metric tons; Peru and Bolivia produced 113.38 (70%) and 44.71 (28%) thousand metric tons, respectively. The remaining 2% is covered by quinoa production in other countries. At the same time, the USA alone imported 30.25 million kg (FAOSTAT, 2025). Today, only two South American countries, Peru and Bolivia, account for more than 98% of the world’s quinoa production and about 70% of global exports. Conversely, quinoa production and research have increased to more than 123 countries (Alandia et al., 2020). It has many genetic materials (mainly in some South American countries), but seed legislation at a global level limits access to genetic resources for testing the crop in new environments (Bazile, 2023). Quinoa is an annual herbaceous crop belonging to the family *Amaranthaceae*, which also includes popular vegetables such as spinach and amaranth. It is a dicotyledonous plant and, therefore, is not cereal, instead known as a pseudocereal. In addition to nutritional qualities, quinoa can grow under diverse agroecological conditions from sea level to an altitude of 3800 m and is tolerant to frost, salinity, and drought (Jensen et al., 2000; Jacobsen et al., 2005; Adolf et al., 2013). Also, quinoa is a low-input crop, ideally suitable for organic and low-input production (De Santis et al., 2016) and can grow in less fertile soil with minimal inputs, such as water and fertilizer.

In addition to quinoa’s grains, its green leaves (quinoa greens) are packed with nutrients and beneficial phytochemicals. However, the consumption of quinoa greens as a vegetable is uncommon. Like grains, leafy greens are rich in nutritional value, boasting higher protein content, lower carbohydrates, and more omega-3 fatty acids than similar leafy vegetables. They are also an excellent source of essential amino acids, vitamins, and minerals. Quinoa greens are higher in protein and lower in carbohydrates than vegetables like spinach and amaranth (El-Samad et al., 2018; Pathan et al., 2019; Pathan and Siddiqui, 2022). Quinoa is drought, salinity-, and cold-tolerant, requiring minimal water and fertilizer to grow. These make quinoa greens an ideal vegetable crop in a climate-changing environment, as they can be grown year-round in fields, greenhouses, or high tunnels, with a harvesting period of about 30 days. The fresh leaves and tender shoots of quinoa can be eaten as cooked vegetables (e.g., stir-fried, sautéed, stewed, and steamed) and as a salad to which young leaves, microgreens, and sprouts can be added (Jancurová et al., 2009; Gawlik-Dziki et al., 2013; Pathan et al., 2019; Vazquez-Luna et al., 2019). In taste surveys, quinoa leafy greens were found to have a taste, texture, and flavor comparable to or even better than spinach (Pathan, personal communication).

Generally, dual-purpose crops (DP) are annual crops such as cereals (e.g., wheat, barley, oat, and triticale) and brassicas (mainly canola) grown to be grazed by livestock during the early vegetative stage and harvested for grains at maturity (McGrath et al., 2021). Other crops, like amaranth, serve as DP crop, with green leaves harvested during early growth stages and grains collected at maturity for human consumption (Hoidal et al., 2019). They found that up to 50% of leaf removal impacted neither seed yield nor quality. Usually, quinoa grows separately to harvest grains, green leaves, and forage (Shah et al., 2020; Pathan et al., 2023a, b; Rubinovich et al., 2023). Based on the results of an earlier field study using quinoa as a DP crop, we found that green leaves and grains can be harvested from the same plants without yield loss. In that case, the main harvest was grains, and the secondary harvest was green leaves. We cut 40-50% of the top portion of the seedlings as green leaves about 4 weeks after seed germination. As far as we know, this study is the first in the USA to use quinoa as a DP crop to determine the feasibility of harvesting quinoa leafy greens and grains from the same plants. Small and limited-resource farmers would benefit from growing DP quinoa, which could provide nutrition from green leaves and grains while maintaining profit from grain production.

### 1.1 Hypothesis and objectives

We hypothesized that quinoa can be grown for dual purposes: leafy greens and grains production and thus enhancing quinoa harvest. To validate this assumption, this study aimed to evaluate the yield and nutritional compositions of quinoa grains of different genotypes between cut and control (uncut) treatments, optimize planting dates, and promote new production techniques among small/limited-resource farmers to increase their farm profitability.

## 2 Materials and methods

### 2.1 Materials

Four quinoa lines, namely, PI614927, PI665275, PI698747, and PI698769 (the origin of the first two are Chile and Bolivia, respectively, and the last two are the USA), were used in this study. These lines were selected based on earlier results of quinoa grains and leafy greens yield performance (Pathan et al., 2023a, b). Seeds were collected from the USDA-ARS Germplasm Resources Information Network (GRIN-North Central Research Plant Introduction Station, Ames, IA, USA) and increased at Lincoln University of Missouri, Jefferson City, MO, USA.

### 2.2 Methods

**Figure 1:**
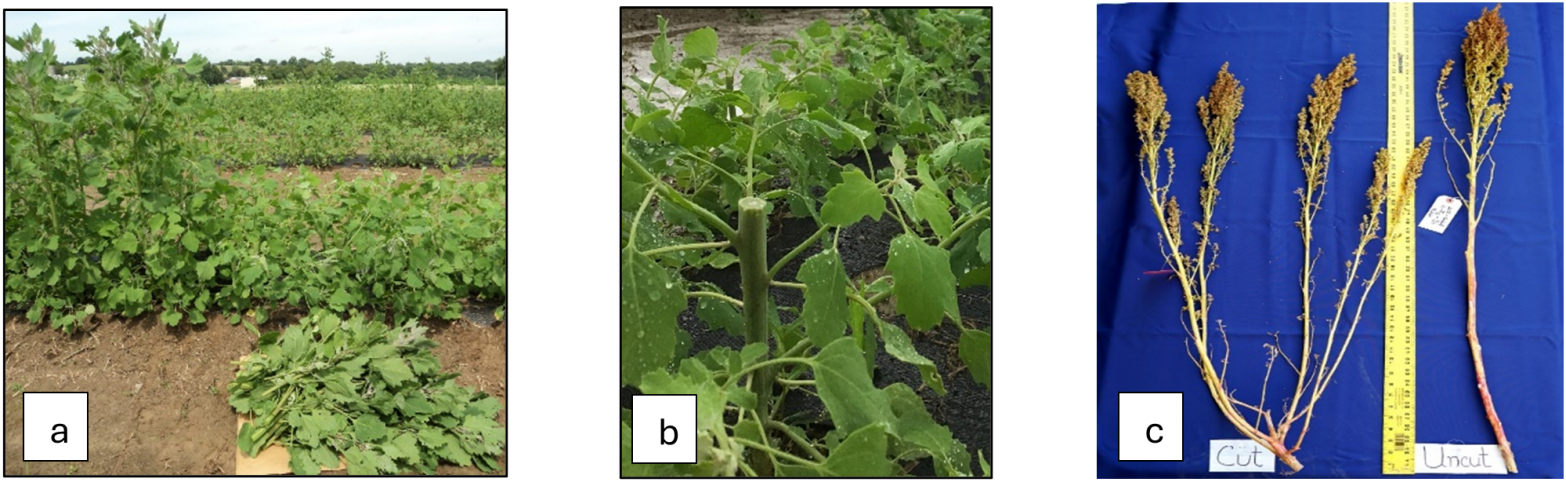
**(a)** Control and cut plants (30DAS), **(b)** a close view of a cut plant with tillering, and **(c)** a mature panicle of cut and control plants.

The research was conducted during the summer of 2024 at the George Washington Carver Farm (lat. 38.32*°* N, long. 92.80*°* W, and elevation 170 m) of Lincoln University of Missouri, Jefferson City, Missouri, USA. The experimental design, field preparation, and planting method were used following the methodology described by Pathan et al. (2023a). All-purpose NPK 12-12–12 fertilizer was applied at a rate of 42 kg per hectare during the land preparation. The experiment was conducted on a raised bed of 33 m long and 0.70 m wide following the design RCB with three replications, which contained a total of 24 sub-plots. The raised bed was covered with a plastic woven weed barrier to control weeds, but it allowed rain and water to penetrate. The net area of each subplot was 1.1 m long and 0.7 m wide, with an area of 0.77 m^2^. The experiment was repeated three times (Date 1, Date 2, and Date 3) at an interval of seven days and started the first planting on May 31 (Date 1), June 6 (Date 2), and June 13 (Date 3). When the seedlings were 4 weeks after germination, green leaves from half of each subplot were harvested about 15–18 cm above the ground (named the ‘cut’ plot) and allowed to grow until maturity. The remaining half of the uncut plot allowed it to grow until maturity (called the ‘control’ plot (**Figure 1**). Normal management practices were followed for both ‘cut’ and ‘control’ plots. Plots were irrigated at 596 liters per hour (L/h) per 100 m or 0.61 L/h per dripper for an hour every two days using a drip irrigation system. When required, weeds were manually removed throughout the growing season (June to September). No herbicide was applied. After flowering, an insecticide called ‘sevin’ (concentration 0.12 L per 3.78 L) was sprayed once to control tarnished plant bugs called lygus bugs (*Lygus lineolaris* L.).

### 2.3 Data collection

#### 2.3.1 Agro-morphological data

The 4-week-old green leaves were harvested about 15–18 cm above ground from the cut plot (harvested area 0.77 square meters). Different agro-morphological data, such as plant height, maturity, shoot dry weight, root dry weight, root length, panicle length, 100-seed weight, and grain yield, were collected from three randomly selected plants of each replication.

#### 2.3.2 Climate data

Air temperature, rainfall, and relative humidity (RH) data (MayS-September, 2024) were collected from the USDA-NRCS National Weather and Climate Center (NWCC)-Soil Climate Analysis Network (SCAN) site #2223 located at George Washington Carver Farm in Jefferson City, MO, USA (https://wcc.sc.egov.usda.gov/nwcc/site?sitenum=2223, accessed on December, 2024). During the cropping season (June to September 2024), the maximum field temperature reached 35.7°C, while the minimum was 11.1°C, with an average temperature of 23.2°C. Total rainfall during that time was 47.10 cm, and the average relative humidity was 90%. The average temperature during the flowering period (the last week of July and the first week of August) was nearly 34°C.

### 2.4 Data analysis

#### 2.4.1 Chemical analysis of quinoa grains

The proximate composition (protein, fat, fiber, ash, and moisture), amino acid, and mineral analyses were performed following Pathan et al. (2023a). The carbohydrate content was estimated by applying the following calculation:

Carbohydrate (%) = 100% - % (crude protein + crude fat + ash + moisture)

The food values of quinoa grains were calculated by multiplying protein, fat, and carbohydrate contents by factors of 4, 9, and 4, respectively, and adding these values to obtain kcal per 100g (Indrayan et al., 2005).

#### 2.4.2 Statistical analysis

A standard statistical procedure was used to evaluate quinoa genotypes grown under two treatment groups (cut and uncut/control) for agro-morphological traits, such as plant height, maturity, shoot dry weight, root dry weight, root length, panicle length, 100-grain weight, yield, and nutritional composition (proximate, amino acid, and mineral). Data were analyzed using SAS statistical software (general linear model-GLM) to realize variability among genotypes and treatment groups for yield, all agronomic traits, and nutritional values (SAS, 2011). Tukey’s honest significance difference (HSD) test was used at the *p* ≤ 0.05 significance level to determine differences in nutritional components for different plant parts. Pearson’s correlation analysis was performed using the metan package in R software.

## 3 Results

### 3.1 Leafy greens

There was a significant difference in leafy greens’ fresh weight for quinoa genotypes studied (data not presented). The average leafy greens yield was about 560g m^2-1^, and the yield was highest (811g) in genotype PI665275 and lowest (339g m^2-1^) in genotype PI698747. The other two genotypes yielded close to the average (around 567g m^2-1^). Date 1 planting conceded the highest yield (684g m^2-1^).

### 3.2 Agro-morphological traits

The analysis of variance (ANOVA) revealed significant (p ≤ 0.05) differences among quinoa genotypes (Var) and between treatments (Trt) for various agro-morphological traits including plant height (Pht), maturity (Mat), shoot dry weight (Sdw), root dry weight (Rdw), root length (Rln), panicle length (Pln), 100-seed weight (Hsw), yield (Yld), stomatal conductance (Stoma), and photosynthetic activity (Photo). In contrast, no significant differences were observed between treatments for proximate composition namely protein (Pro), fat (Fat), fiber (Fib), ash (Ash), and carbohydrate (Carb); all essential amino acids, histidine (His), isoleucine (Ile), leucine (Leu), lysine (Lys), methionine (Met), phenylalanine (Phe), threonine (Thr), tryptophan (Trp), and valine (Val); and minerals, calcium (Ca), potassium (K), magnesium (Mg), phosphorus (P), iron (Fe), and zinc (Zn) (**Supplementary Table S1**).

A highly significant difference (*P*<0.001) was found for Trt among Pht, Mat, Sdw, Pln, and Yld. Also, a significant difference was observed among quinoa genotypes for Pht, Mat, Rdw, Rln, and Pln. However, the Var x Trt interaction was insignificant for all the studied traits, indicating that Var showed stability across the studied traits. Additionally, there was no significant difference between Var, Trt, and Var x Trt for Hsw (**Supplementary Table S1**).

The average value of different agro-morphological traits of four quinoa genotypes grown under two treatments is shown in **Table 1**. Quinoa plant height (Pht) was significantly higher in the control (132 cm) than in the treatment (113 cm). The mean Pht under the two treatments ranged from 117–144 cm, and 109–118 cm in control and cut plants, respectively. The days to maturity (Mat) of quinoa genotypes showed a significant difference for Var, Dop, and Trt (**Supplementary Table S1**). The Mat ranged from 87 to 92 days and was higher in cut treatment (90 days) than the control (88 days) (**Table 2**).

**Table 1:** Least square means of treatment (cut and control) (n=48) ± standard error (SE) and ranges of different nutritional components of quinoa.

Traits	Treatment means		Ranges		^1^Published results
	Cut	Control	Cut	Control	Ranges (min-max)
Proximate (g 100^-1^g DW)					
Protein (Pro)	15.18 ±0.17	15.05 ±0.37	14.64-15.39	14.90-15.74	9.10-15.70
Fat (Fat)	5.63 ±0.06	5.51 ±0.10	5.44-5.82	5.47-5.62	.00-7.60
Fiber (fib)	2.71 ±0.01	2.71 ±0.10	2.55-2.92	2.57-2.82	1.00-9.20
Ash (Ash)	3.75 ±0.07	3.72 ±0.07	3.67-3.84	3.53-3.80	2.00-7.70
Carbohydrate (Carb)	65.27 ±0.21	65.32 ±0.43	64.93-65.67	64.42-66.27	48.50-69.80
Amino Acids (g 100^-1^g protein)					
Histidine (His)	2.59 ±0.02b	2.64 ±0.03a	2.55-2.62	2.61-2.66	1.40-4.40
Isoleucine (Ile)	3.57 ±0.03	3.57 ±0.04	3.58-3.67	3.56-3.61	0.80-7.40
Leucine (Leu)	5.52 ±0.03	5.53 ±0.04	5.45-5.55	5.48-5.59	2.30-9.40
Lysine (Lys)	5.18 ±0.04	5.18 ±0.05	5.15-5.23	5.12-5.20	2.40-2.70
Methionine (Met)	1.73 ±0.01	1.72 ±0.02	1.71-1.73	1.70-1.74	0.30-9.10
Phenylalanine (Phe)	3.64 ±0.03	3.67 0.05	3.61-3.65	3.63-3.72	2.70-10.30
Threonine (Thr)	3.09 ±0.02	3.11 ±0.02	3.03-3.11	3.08-3.13	2.10-8.90
Tryptophan (Trp)	0.93 ±0.01	0.93 0.01	0.91-0.95	0.93-0.95	0.60-1.90
Valine (Val)	4.09 ±0.02	4.11 ±0.03	0.91-0.95	0.93-0.95	0.60-1.90
Minerals (mg 100^-1^g DW)					
Calcium (Ca)	76.40 ±2.27	78.99 ±2.50	72.25-79.10	70.78-90.52	28.00-149.00
Potassium (K)	1363 ±27.86	1352 ±19.03	1290-1465	1295-1380	656-1475
Magnesium (Mg)	273 ±5.21	270 ±4.12	183-273	264-280	207-502
Phosphorus (P)	477 ±8.76	491 ±7.20	477-482	482-504	350-482
Iron (Fe)	6.25 ±0.29	6.25 ±0.29	5.58-6.81	5.64-7.33	2.60-15.00
Zinc (Zn)	4.86 ±0.12	5.08 ±0.15	4.32-5.16	4.55-5.42	0.79-4.00

^1^ Nowak et al., 2016.

Different letters suggest a significant difference between treatment means (cut and control) in the same row indicated by Tukey’s honestly significant difference (HSD) test at *p ≤*0.05. All value pairs showed non-significant (ns) except for histidine, and DW dry weight.

**Table 2:** Least square means of treatment (cut and control) (n=72) ± standard error (SE) and ranges of different agro-morphological traits of quinoa.

Traits	Treatment means		Ranges (Min-Max)	
	Cut	Control	Cut	Control
Plant height (Pht, cm)	113.11 ±2.74b	131.95 ±2.71a	109.08-118.63	116.89-143.99
Maturity (Mat, days)	90 ±0.81a	88 ±0.81b	87-92	87-91
Shoot dry weight (Sdw, g)	77.67 ±4.72a	53.80 ±3.26b	70.60-87.68	47.31-60.12
Root dry weight (Rdw, g)	14.99 ±1.12a	12.87 ±0.89b	12.28-17.41	9.81-15.08
Root length (Rln, cm)	20.80 ±0.95ns	21.28 ±1.71ns	18.77-24.55	18.77-14.62
Panicle length (Pln, cm)	32.46 ±0.88b	38.03 ±0.80a	29.40-35.13	36.00-39.31
100-seed weight (Hsw, g)	0.23 ±0.004ns	0.23 ±0.004ns	0.22-0.24	0.23-0.23
Yield (Yld, g/plant)	22.87 ±1.76a	15.52 ±1.46b	20.02-29.16	12.17-18.10
Total biomass (Bio, g/plant)	92.67 ±5.55a	66.67 ±3.85b	83.65-104.92	60.90-73.10
Harvest index (Hi, %)	25.42 ±1.70ns	23.22 ±1.58ns	22.41-27.95	19.36-27.70

Different letters suggest a significant difference between treatment means (cut and control) in the same row indicated by Tukey’s honestly significant difference (HSD) test at *p ≤*0.05, ns not significant.

The shoot dry weight (Sdw) displayed a significant difference for Dop and Trt (**Supplementary Table S1**) but not for Var. The average Sdw for cut and control was 77.67 and 53.80 g plant^-1^, respectively; that is, cut plants have higher Sdw than the control. The root dry weight (Rdw) exhibited a significant difference for Var and Dop (**Supplementary Table S1**). The average Rdw for cut and control was 14.99 and 12.87g plant^-1^, respectively. The cut plants have higher Rdw than the control. Although quinoa genotypes showed a difference in root length (Rln), ranging from 18.77-24.62 cm, the difference was insignificant between cut (20.80 cm) and control (21.28 cm) treatments (**Table 2**).

A significant difference was found for Var, Dop, and Trt with panicle length (Pln), which varied between the treatments: 32.46 cm for cut and 38.03 cm for control. All genotypes in the control treatments exhibited a higher Pln than the genotypes in the cut. No variability was observed among quinoa genotypes and between the treatments for 100-seed weight (Hsw). The HSW ranged from 0.22 to 0.24 g. A significant difference (*P <*0.001) was observed for Dop and Trt with yield (Yld), but there was no difference for Var. Yld difference varied between cut and control treatments, 22.87 g (range 20.02-29.16 g) and 15.52 g (range 12.17-18.10 g) plant^-1^, respectively (**Table 2**). The highest yield (29.16 g plant^-1^) was recorded for genotype PI614927 in the cut treatment, and the same genotype yielded 18.10 g in the control.

Plant biomass (Bio) differed significantly in Trt. The cut treatment had a higher total biomass (92.67g plant^-1^, ranging between 104.92 and 83.65g) than the control (66.67g plant^-1^, ranging between 73.10 and 60.90g).

The harvest index (Hi), one of the most critical components of grain yield, was calculated as the ratio between quinoa grain yield and total biomass (stems, panicles, and roots) expressed in percentage. The cut treatment had a higher Hi, 0.25 (ranging between 0.22 and 0.28), than the control, 0.23 (ranging between 0.19 and 0.28), but the difference was insignificant (**Table 2**).

### 3.3 Nutritional composition of quinoa grains

The proximate analysis (protein, fat, fiber, ash, and carbohydrate) indicated insignificant differences between the genotypes, treatments, and planting dates (**Supplementary Table S1**). The difference between cut and control treatments for protein 15.18 and 15.05g, fat 5.63 and 5.51g, fiber 2.55 and 2.92 g, ash 3.67 and 3.84 g, and carbohydrates 64.93 and 65.67g 100 g^-1^ DW in cut and control plants, respectively (**Table 1**).

All essential amino acids displayed an insignificant difference between the treatments (**Table 1**). The mean values (g 100 g^-1^ protein) between the cut and the control treatments were His 2.59 and 2.64, Ile 3.57 and 3.57, Leu 5.52 and 5.53, Lys 5.18 and 5.18, Met 1.73 and 1.72, Phe 3.64 and 3.67, Thr 3.09 and 3.11, Trp 0.93 and 0.93 and Val 4.09 and 4.11g 100g^-1^ protein, respectively (**Table 1**).

The mineral elements (Ca, K, Mg, P, Fe, and Zn) showed no significant difference between the cut and control treatments (**Table 1**). The mean values (mg 100 g^-1^ DW) between the cut and control treatments as Ca (76.40 vs.78.99), K 1363 vs. 1352, Mg (273 vs 270), P (477 vs491), Fe (6.25 vs. 6.25), and Zn (4.86 vs. 5.08), respectively (**Table 1**).

In this study, significant relationships among various nutritional components were observed. For example, protein (Pro) was positively correlated with magnesium (Mg) (r = 0.42) and negatively correlated with carbohydrate (Carb) (r = –0.76) and methionine (Met) (r = –0.70). Date of planting showed significant positive correlations with Met (r = 0.35), tryptophan (Trp) (r = 0.69), lysine (Lys) (r = 0.78), threonine (Thr) (r = 0.70), histidine (His) (r = 0.84), and phenylalanine (Phe) (r = 0.92). Calcium (Ca) was positively associated with potassium (K). The Carb correlates negatively with days of planting (r=-0.41) and showed a non-significant correlation with Trt but a positive relation with Met (r=0.39). Treatment (Trt; cut vs control) did not have a significant impact on the nutritional quality of quinoa genotypes grown under the two treatment conditions and three planting dates during the study period.

## 4 Discussion

Conventionally, quinoa has been cultivated as a monocrop for grains or leafy greens as vegetables or forage (Asher et al., 2020; Shah et al., 2020; Pathan et al, 2023a, b). This research uncovered a new dual-purpose quinoa production technique for harvesting leafy greens first and grains later from a single plant to maximize quinoa production with economic return. This study evaluated four quinoa genotypes under two treatments: ‘cut,’ harvesting leafy greens four weeks after seed germination, and allowing them to grow till maturity, and other ‘control’ (uncut) plants were grown alongside. In addition to leafy greens, a significantly higher grain yield was observed in cut plants than in control plants.

The plant height showed a significant difference between the two treatments. Pht was significantly higher in the control (134 cm) than in the cut (113 cm) because, in the cut plots, plant growth was paused after harvesting leafy greens, allowing side branches to grow. The Pht of individual genotypes followed a similar order between the treatments, and there were no differences in planting dates. Our result, obtained under control treatment, is close to the mean plant height in the United States (Craine et al., 2023; Pathan et al., 2023a). A significant positive correlation was found between Pht and Pln and Rdw, and a negative association was found between Pht and Trt (**Figure 1**).

Plants of the cut treatment took 3–4 more days to mature than control plants (**Table 2**). After harvesting leafy greens (plant growth was delayed due to harvesting leafy greens), cut plants took several days to grow new branches and finally delayed maturity. Meanwhile, control plants continued normal growth to maturity. Quinoa’s photoperiod sensitivity character made the difference between the treatments only a few days. Yld, Sdw, and Rdw displayed a significant positive correlation with Mat and a negative association with Dop (**Figure 1**).

**Figure 2:**
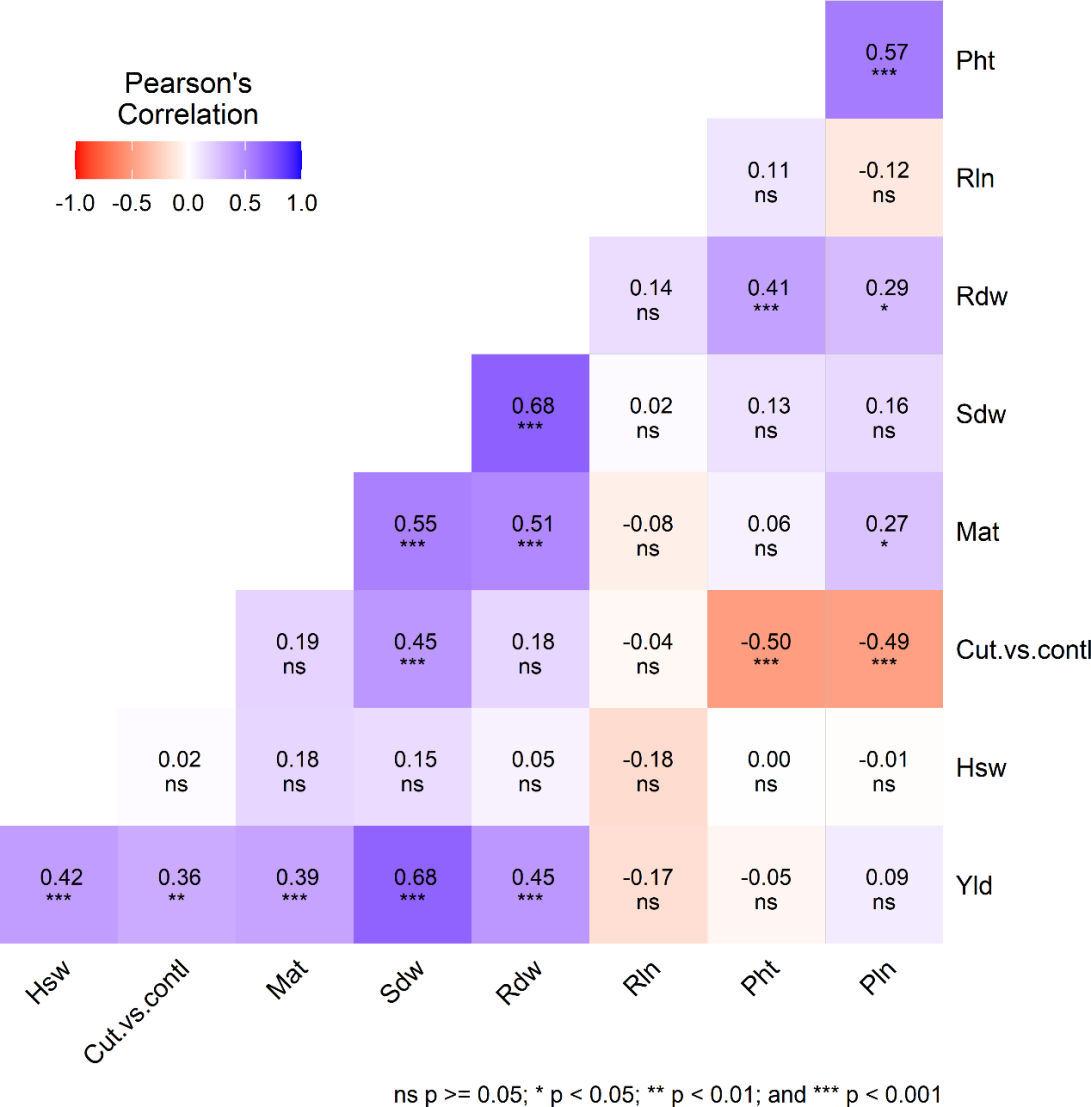
Pearson’s correlation coefficient (r) illustrates the relationships among various agro-morphological traits, including plant height (Pht), root length (Rln), root dry weight (Rdw), shoot dry weight (Sdw), maturity (Mat), treatment (cut vs. control), hundred seed weight (Hsw), and grain yield (Yld) among quinoa genotypes cultivated under two treatments (cut and control) and three planting dates in 2024.

In the control plot, only one undisturbed shoot attained maturity. However, in the cut plot, several branches appear (on average, four per plant) after harvesting the leafy greens and reaching maturity (**Figure 1**). The Sdw represents only one shoot in the control plot, but the combination of several smaller shoots denotes a higher Sdw in the cut plot. Trt, Mat, Yld, and Rdw showed a significant positive correlation with Sdw and a negative association with Dop. Rdw showed a positive correlation with Pht, Mat, Pln, Yld, and Rdw and a negative association with Dop (**Figure 2**).

Root length (Rln) showed no significant difference among genotypes, and the difference between the cut and control treatments also showed no significance. Additionally, no significant correlations were observed among the traits studied except for Dop (*P*=0.004).

The Pln was significantly higher among genotypes and between the control plants than cut because cut plants had shorter multiple-branch panicles than single-branch panicles with larger panicles in control plants. Pln was positively correlated with Pht (*P*<0.0001) but negatively correlated with Trt (*P*<0.0001), indicating that control treatment signifies higher Pln. The panicle length in this study was closer to what was reported by Craine et al. (2023) but higher than that of Pathan et al. (2023a).

There was an insignificant difference in Hsw between the genotypes and treatments, indicating no effect of cut treatment on seed weight. The Hsw in this study was lower than Craine et al. (2023) reported, but closer to what Pathan et al. (2023a) reported.

This study found a significant variation in grain yield for genotypes and treatments. Grain yield showed a significant positive correlation with Trt, Mat, Hsw, Sdw, and Rdw, but a negative association with Dop. Our study agrees with earlier findings of the correlation of grain yield with Mat and Hsw (Craine et al., 2023; Pathan et al., 2023a). This finding cannot be directly compared to other results due to substantial variations in yield and yield-related traits found in different countries, as well as the use of different genotypes, test locations, soil, temperature, humidity, rainfall, treatments (dual-purpose use), and altitude (Pathan et al., 2023a). Moreover, a significant positive correlation was found in grain yield with Sdw, Rdw, Mat, and Hsw. Cut plants accumulated higher root and shoot masses than the control plants. Higher shoot and root mass positively contributed to grain yield. In contrast, root length had an insignificant correlation with yield and yield contributing traits.

A significantly higher amount of total biomass (Bio) contents in cut plants was due to a higher number of tillers in the cut plants, showing a positive correlation with grain yield. Tang et al. (2024) suggested that high-yielding varieties allocate more resources to biomass production, increasing yield potential. The harvest index (Hi) reflects the photosynthetic ability of plants to convert seed production capacity (Bertero and Ruiz, 2010; Tang et al., 2024). A significant positive correlation between Yld and Hi was found in this study, which agrees with earlier studies. The quinoa harvest index is reported to range from 0.06 to 0.87 (Rojas, 2003) and 0.10 to 0.55 (Maliro et al., 2017). In the current study Hi ranged from 0.22 to 0.28, which falls within the range of earlier reports.

**Figure 3:**
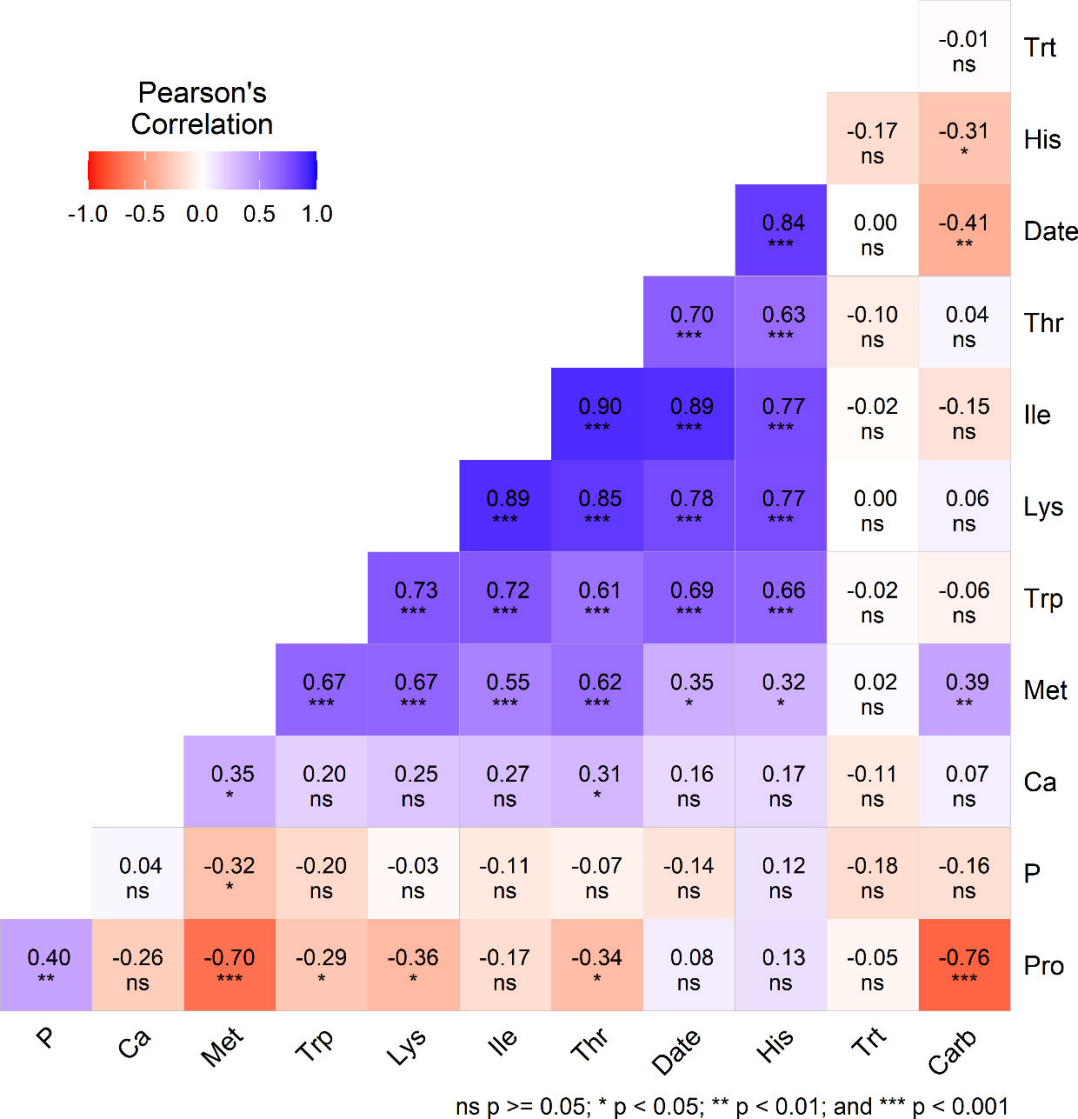
Pearson’s correlation coefficient (r) illustrates the relationships among different nutritional components, such as protein (Pro), magnesium (Mg), methionine (Met), tryptophan (Trp), lysine (Lys), threonine (Thr), histidine (His), date of planting (Date), phenylalanine (Phe), calcium (Ca), potassium (K), and treatment (trt, cut and control) among quinoa genotypes grown under two treatments (cut and control) and three planting dates during cropping season.

There was no significant difference in protein, fat, fiber, ash, and carbohydrate amounts between the two treatments (**Table 2**), suggesting that the cut treatment had no impact on the grain’s proximate composition compared to the control. All essential amino acids, except for histidine, showed no significant difference between the treatments (**Table 1**). These results indicate that the amino acids produced in the grain of cut plants are like those of the control, suggesting that cutting has no effect on amino acid concentrations. Amino acid concentrations in this study agree with previous findings (Nowak et al., 2016; Pathan et al., 2023a). Similarly, the mineral elements (Ca, K, Mg, P, Fe, and Zn) showed no significant difference between the treatments (**Table 1**). This implies that the mineral concentration in the grain of cut plants is like that of the control plants, indicating that cutting has no effect on mineral concentrations. The results of this study agree with previous findings (Nowak et al., 2016; Pathan et al., 2023a). The negative correlation between Pro and Carb observed in this study supports previous findings (Pathan et al., 2024). The non-significant correlation between protein and treatment (Cut vs control) or days of planting suggests that these variables have no measurable effect on protein quality (**Figure 3**).

## 5 Conclusion

The results showed that quinoa can be grown as a dual-purpose crop to maximize production with economic and environmental benefits. This new quinoa production technique demonstrated that leafy greens can be harvested at the early stage of growth as green vegetables and grains from the same plant at maturity for human consumption. In addition to leafy greens, more quinoa grains were harvested from the cut plants than control. There were no significant differences in grain nutritional composition between the cut and control treatments. Among the three planting dates, early planting showed a higher harvest of greens and grains, indicating its potential as a practical production technique. Further research is needed to explore maximizing yield across the locations and environments. To achieve this, we have planned a complete study under three environments, i.e., drought, rainfed, and control (irrigated), and at numerous locations.

## Data Availability

The original contributions presented in the study are included in the article/**Supplementary Material**. Further inquiries can be directed to the corresponding author.
