# Supplementary material for: Maximizing quinoa production through a dual-purpose harvesting method
Source: Front Plant Sci. 2025 Jul 16;16:1606163. doi: 10.3389/fpls.2025.1606163 (PMC12307409; doi:10.3389/fpls.2025.1606163)
Supplement: Supplementary file 1 [file Table1.docx]

**Supplemental Table S1**. The analysis of variance (ANOVA) of agro-morphological traits, proximate components, amino acids, and minerals.

Parameter Source DF F ratio Prob> F

**Agro-morphological**

Pht Var 3 3.59 0.02*

Dop 2 0.15 0.86

Trt 1 22.56 <0.0001***

Var x Dop 6 0.21 0.97

Var x Trt 3 1.00 0.40

Mat Var 3 9.40 <0.0001***

Dop 2 76.91 <0.0001***

Trt 1 9.33 0.003**

Var x Dop 6 0.38 0.89

Var x Trt 3 3.20 0.0.04*

Sdw Var 3 1.44 0.24

Dop 2 10.14 0.0002***

Trt 1 19.42 <0.0001***

Var x Dop 6 0.33 0.92

Var x Trt 3 0.08 0.97

Rdw Var 3 2.94 0.04*

Dop 2 7.83 0.001***

Trt 1 2.59 0.11

Var x Dop 6 0.32 0.92

Var x Trt 3 0.59 0.64

Rln Var 3 2.96 0.04*

Dop 2 4.71 0.02*

Trt 1 0.12 0.73

Var x Dop 6 0.67 0.68

Var x Trt 3 0.44 0.72

Pln Var 3 4.06 0.01**

Dop 2 9.40 0.0004***

Trt 1 28.68 <0.0001***

Var x Dop 6 1.20 0.32

Var x Trt 3 1.01 0.40

Hsw Var 3 0.45 0.71

Dop 2 0.21 0.81

Trt 1 0.03 0.85

Var x Dop 6 1.04 0.41

Var x Trt 3 0.59 0.62

Yld Var 3 2.23 0.10

Dop 2 10.02 0.0002***

Trt 1 12.84 0.0008***

Var x Dop 6 1.03 0.41

Var x Trt 3 0.58 0.63

Stoma Var 3 0.83 0.48

Dop 2 3.08 0.05*

Trt 1 1.61 0.21

Var x Dop 6 1.76 0.11

Var x Trt 3 0.37 0.78

Photo Var 3 0.07 0.98

Dop 2 28.11 <0.0001***

Trt 1 1.04 0.31

Var x Dop 6 0.56 0.76

Var x Trt 3 0.93 0.43

**Proximate**

Pro Var 3 0.30 0.82

Dop 1 0.23 0.62

Trt 1 0.09 0.77

Var x Dop 3 0.11 0.95

Var x Trt 3 0.32 0.81

Fat Var 3 0.19 0.90

Dop 1 0.42 0.52

Trt 1 1.08 0.31

Var x Dop 3 2.39 0.89

Var x Trt 3 0.94 043

Fib Var 3 0.87 0.46

Dop 1 2.26 0.14

Trt 1 0 0.99

Var x Dop 3 0.42 0.74

Var x Trt 3 0.14 0.93

Ash Var 3 1.88 0.15

Dop 1 0.02 0.90

Trt 1 0.28 0.60

Var x Dop 3 1.37 0.27

Var x Trt 3 0.55 0.65

Carb Var 3 0.54 0.66

Dop 1 9.46 0.004**

Trt 1 0.01 0.91

Var x Dop 3 0.88 0.46

Var x Trt 3 1.30 0.29

**Amino Acids**

His Var 3 0.70 0.56

Dop 1 153.10 <0.0001***

Trt 1 5.99 0.02*

Var*Dop 3 0.30 0.83

Var*Trt 3 1.18 0.33

Ile Var 3 0.84 0.48

Dop 1 158.96 <0.0001***

Trt 1 0.08 0.77

Var*Dop 3 0.29 0.83

Var*Trt 3 0.25 0.86

Leu Var 3 1.49 0.23

Dop 1 78.76 <0.0001***

Trt 1 0.14 0.71

Var*Dop 3 0.60 0.62

Var*Trt 3 0.17 0.91

Lys Var 3 0.38 0.77

Dop 1 71.17 <0.0001***

Trt 1 0 0.99

Var*Dop 3 0.39 0.76

Var*Trt 3 0.36 0.77

Met Var 3 0.31 0.82

Dop 1 5.24 <0.0001***

Trt 1 0.02 0.89

Var*Dop 3 0.43 0.74

Var*Trt 3 0.02 0.99

Phe Var 3 1.79 0.17

Dop 1 307.73 <0.0001***

Trt 1 1.52 0.23

Var*Dop 3 0.20 0.90

Var*Trt 3 0.20 0.89

Thr Var 3 1.43 0.25

Dop 1 41.08 <0.0001

Trt 1 0.79 0.38

Var*Dop 3 0.49 0.72

Var*Trt 3 0.34 0.80

Trp Var 3 0.19 0.90

Dop 1 39.18 <0.0001***

Trt 1 0.01 0.91

Var*Dop 3 0.47 0.70

Var*Trt 3 1.08 0.37

Val Var 3 1.41 0.26

Dop 1 35.14 <0.0001***

Trt 1 0.24 0.63

Var*Dop 3 0.45 0.72

Var*Trt 3 0.94 0.43

**Minerals**

Ca Var 3 3.17 0.04*

Dop 1 1.46 0.24

Trt 1 0.71 0.41

Var x Dop 3 0.28 0.84

Var x Trt 3 1.10 0.36

K Var 3 2.69 0.06

Dop 1 0.30 0.59

Trt 1 0.15 0.70

Var x Dop 3 0.35 0.79

Var x Trt 3 1.51 0.23

P Var 3 0.44 0.73

Dop 1 0.83 0.37

Trt 1 1.37 0.25

Var x Dop 3 0.11 0.95

Var x Trt 3 0.09 0.97

Mg Var 3 1.20 0.32

Dop 1 1.65 0.21

Trt 1 0.18 0.67

Var x Dop 3 0.24 0.86

Var x Trt 3 0.47 0.70

Fe Var 3 1.09 0.37

Dop 1 0.13 0.72

Trt 1 0.01 0.96

Var x Dop 3 0.27 0.85

Var x Trt 3 1.32 0.29

Zn Var 3 3.10 0.04*

Dop 1 0.02 0.90

Trt 1 1.02 0.32

Var x Dop 3 0.94 0.43

Var x Trt 3 0.05 0.97

_____________________________________________________________________________________________________

*, **, *** significant differences at *p* <0.05, *p* <0.01, and *p* <0.001, respectively.

**agro-morphological traits**, plant height-Pht, maturity-Mat, shoot dry weight-Sdw, root dry weight-Rdw, root length-Rln, panicle length-Pln, 100-seed weight-Hsw, yield-Yld, stomatal conductance-Stoma, and photosynthetic rate-Photo; **proximate components**, protein-Pro, at-Fat, fiber-Fib, ash-Ash, and carbohydrates-Carb; **amino acids**, histidine-His, isoleucine-Ile, Leucine-Leu, lysine-Lys, methionine-Met, phenylalamine-Phe, threonine-Thr, tryptophan-Trp, and valine-Val; **minerals**, calcium-Ca, potassium-K, Phosphorus-P, magnesium-Mg, Iron-Fe, and Zinc-Zn.
